# Novel Experimental Methods for the Investigation of *Hermetia illucens* (Diptera: Stratiomyidae) Larvae

**DOI:** 10.1093/jisesa/ieaa057

**Published:** 2020-06-27

**Authors:** Moritz Gold, Melanie Binggeli, Fabienne Kurt, Tomas de Wouters, Markus Reichlin, Christian Zurbrügg, Alexander Mathys, Michael Kreuzer

**Affiliations:** 1 ETH Zurich: Swiss Federal Institute of Technology Zurich, Laboratory of Sustainable Food Processing, Zurich, Switzerland; 2 Eawag: Swiss Federal Institute of Aquatic Science and Technology, Department Sanitation, Water and Solid Waste for Development (Sandec), Dübendorf, Switzerland; 3 PharmaBiome AG, Zurich, Switzerland; 4 ETH Zurich, Institute of Agricultural Sciences, Zurich, Switzerland

**Keywords:** insect, digestion, microorganism, sterile, frass collection

## Abstract

Large-scale insect rearing for food and feed production can be improved by understanding diet digestion and host–microbe interactions. To examine these processes in black soldier fly (*Hermetia illucens* L.; Diptera: Stratiomyidae) larvae, two protocols were developed. Protocol 1 describes a method to produce viable, sterile black soldier fly larvae and a gentle method for diet sterilization. Sterile black soldier fly larvae can be used to study the diverse role of microbes in larval development. Nutrient requirements of sterile black soldier fly larvae are met only through diet. Viable sterile black soldier fly larvae were consistently generated using a four-step treatment with alternating immersions of eggs for 2 min each in ethanol (70%) and sodium hypochlorite (0.6%), over two cycles. A nonthermal method of diet sterilization, namely high-energy electron beam (HEEB) treatment, was introduced. Subsequently, growth of sterile black soldier fly larvae was observed on the HEEB-treated diets (40, 60, and 40% of replicates with poultry feed, liver pie, and an artificial diet, respectively) but not on autoclaved diets. In Protocol 2, we propose a novel method to collect frass from individual larvae. We then measured the metabolites in frass, using high-pressure liquid chromatography. Results on metabolites confirmed the influence of digestion. For instance, succinate increased from 1 to 2 and 7 μmol/g sample from diet to gut homogenate and frass, respectively. The collection method is a promising tool to estimate the diet and nutrient requirements of black soldier fly larvae, thus increasing the performance and reliability of black soldier fly larvae rearing. We discuss in detail the possible applications and limitations of our methods in black soldier fly larvae research.

Upcycling of organic wastes (e.g., animal manure, food waste) and side streams (e.g., food industry by-products) by insects (Zurbrügg et al. 2018) for sustainable food and feed production requires efficient large-scale rearing facilities (Smetana et al. 2019). Currently, larvae of the black soldier fly (*Hermetia illucens* L. (Diptera: Stratiomyidae)) are among the most promising insects reared as animal feed (Pinotti et al. 2019; Bosch et al. 2020) and food (Barroso et al. 2017). Extensive research has been conducted on the influence of dietary nutrients on the growth of black soldier fly larvae (Lalander et al. 2013; Nguyen et al. 2013; Barragán-Fonseca et al. 2017, 2018; Cammack and Tomberlin 2017; Spranghers et al. 2017; Gold et al. 2020). Process reliability is a major challenge in large-scale rearing of insects for animal feed markets. Current process performance (e.g., larval growth, bioconversion rate) is often low or varies between diets (Lalander et al. 2018, Nyakeri et al. 2019, Gold et al. 2020), thus affecting day-to-day operations and sustainability (Smetana et al. 2019). It is therefore necessary to determine the specific nutrient requirements of black soldier fly larvae as well as their nutrient utilization.

In order to thrive, insects depend on their gut microbiota, as these microbes degrade food constituents to products that can be absorbed in the gut (Douglas 2010, Engel and Moran 2013). In addition, they produce compounds and metabolites that are either essential for the organism’s metabolism (Shin et al. 2011; Storelli et al. 2011, 2018) or play a role in immune response (Broderick and Lemaitre 2012) and detoxification of contaminants (e.g., secondary plant metabolites, mycotoxins) (Fusetto et al. 2017). Black soldier fly larvae harbor a rich and diverse microbial community (Bruno et al. 2019). The addition of bacteria to diets has further increased rearing performance (Somroo et al. 2019). However, the role of microbiota in black soldier fly larvae and their application in mass-rearing of this insect is yet poorly understood (De Smet et al. 2018); although several similarities may be derived from extensive research on the *Drosophila melanogaster* (Diptera: Drosophilidae) model (Shin et al. 2011; Storelli et al. 2011, 2018; Gold et al. 2018). These processes have been examined across several disciplines in sterile organisms. Numerous attempts have been made to sterilize insect eggs (Table 1). Sodium hypochlorite is an especially promising chemical that induces egg sterilization by removing the egg chorion (Broderick and Lemaitre 2012). Egg sterilization is well established in several insect species, especially *Drosophila* spp. However, in existing literature (Table 1) a large variation is observed in the concentration and combinations of chemicals used, such as ethanol and formaldehyde, as well as the number of egg immersion cycles. In addition, no papers have been published on successful rearing of sterile black soldier fly larvae that can possibly be used as a research model system. It is difficult to achieve maximal hatching rates at complete egg and larval sterility, since both sterility success and mortality increase with increasing concentration of the sterilizing chemical and duration of exposure. Moreover, since black soldier fly eggs are oviposited in clusters of several hundred eggs, it is necessary to separate them to allow the sterilizing chemicals to treat the entire surface area of the egg without destroying the inner layer that carries the larva. Results of our preliminary experiments as described in the first protocol below indicate that currently established methods for production of sterile *Drosophila* spp. are unable to produce sterile and growing black soldier fly larvae.

**Table 1.** Established sterilization methods for the production of sterile insects, as described in literature

Insect species	Method	Reference
*Dacus dorsalis* Hendel (Diptera: Tephritidae)	Immersion in a solution with 10% formaldehyde and 0.2% hydrochloric acid for 10 min, followed by four washing cycles with sterile, double distilled water	Tamashiro et al. (1990)
*Drosophila* spp.	Immersion in 2.7% sodium hypochlorite for 2 min, two washing cycles with 70% ethanol, followed by two washing cycles with sterile, distilled water	Brummel et al. (2004)
*Drosophila melanogaster*	Immersion in 10% sodium hypochlorite for 5 min, rinsed three times with sterile water	Ridley et al. (2012)
*Drosophila melanogaster*	Three washing cycles with 0.6% sodium hypochlorite, followed by three washing cycles with sterile water	Newell and Douglas (2014)
*Drosophila melanogaster*	Immersion in 2.7% sodium hypochlorite for 2–3 min, followed by two washing cycles with 70% ethanol and three washing cycles with sterile water for 10 min	Sabat et al. (2015)
*Drosophila melanogaster*	Two washing cycles with 0.6% sodium hypochlorite for 2.5 min each, followed by three washing cycles with sterile water	Koyle et al. (2016)
*Drosophila melanogaster*	Three washing cycles with 0.6% sodium hypochlorite, followed by three rinsing cycles with sterile water	Kim et al. (2018)
*Drosophila melanogaster*	Immersion in 1% active chlorine (50% bleach) for 5 min, two washing cycles with 70% ethanol for 1 min and sterile water for 1 min	Kietz et al. (2018)
*Lucilia sericata* (Diptera: Calliphoridae)	Immersion in 0.05% sodium hypochlorite for 5 min, followed by immersion in 5% formaldehyde for 5 min, washed with sterile distilled water	Mumcuoglu et al. (2001)
*Musca domestica* L. (Diptera: Muscidae)	Immersion in 0.26% sodium hypochlorite for 25 min, followed by three washing cycles with sterile water	Schmidtmann and Martin (1992)
*Hermetia illucens*	Three washing cycles with 70% ethanol followed by three washing cycles with sterile water	Wynants et al. (2019)

As in livestock, dietary nutrients (e.g., protein, carbohydrates) are essential for the development of insects (Danielsen et al. 2013). Information on both nutrient composition and digestibility of the diet is required to determine diet quality. This information also facilitates calculation of both the protein/amino acid and energy requirements of black soldier fly larvae according to their developmental stage (i.e., hatchlings vs late-instar larvae), environment (e.g., temperature), and operating conditions (e.g., feeding depth affecting oxygen supply). With this knowledge, diets with an optimal nutrient composition can be provided in the ideal amounts. Digestibility measurements determined by mass balance calculations between nutrients in food/feed and human/animal feces, using the nutritional value of individual foods/feeds described in databases, are the current standard in human and livestock nutrition. Similar methods may be implemented to obtain information on the digestibility of black soldier fly larvae diets. When combined with growth assessment methods, they can provide information on nutrient requirements of black soldier fly larvae. However, to the best of our knowledge, there has not yet been any method describing the collection of frass from black soldier fly larvae. As these larvae naturally live within their diet, they consume from and excrete into the same environment. The resulting residue is therefore a mixture of unconsumed diet and frass, and cannot be completely separated into its constituent fractions. Few methods of insect frass collection have been reported so far. Hagley (1962) constructed a collection cage and Sharma (1973) elucidated a method in which whole insects were fixed in wax. In both methods, the insects were immobilized, which likely affected their behavior and diet intake. Pathak et al. (1982) developed a parafilm sachet method covering insects living on a plant, where frass can be clearly distinguished from dietary plant material. This is not possible with black soldier fly larvae. Therefore, frass collection in black soldier fly larvae is currently limited to indirect methods such as measurement of waste reduction, and larval growth and composition (Gold et al. 2020). Waste reduction underestimates the amount of diet ingested, since diet residue and frass are analyzed together. Data on larval growth and composition aggregate several digestive and metabolic processes. It therefore hampers identification of the effect of individual nutrients and whether growth was limited by other nutritional (e.g., imbalanced protein-energy ratio) or nonnutritional factors (e.g., nutrient supply exceeds the genetically predetermined growth potential). Other approaches, such as collecting entire gut contents from individual larvae, include both digested as well as undigested contents, thus making it impossible to accurately measure the effectively digested diet portion.

The objective of this study was to develop protocols that will help researchers to better understand the role of gut microbiota and diet nutrient requirements in black soldier fly larvae. First, we established a method that successfully generated sterile larvae in Protocol 1. Subsequently, we hypothesized that microbes provide nutrients that are essential for larval development. Protocol 1 also describes a method to sterilize eggs and enable larval development, even in the absence of diet- and larva-associated microbes. In Protocol 2, we constructed and tested a device to collect frass from individual larvae. The frass collected using our device is free from diet residues.

## Protocol 1: Establishment of Sterile, Viable, and Growing Black Soldier Fly Larvae

### Experimental Design and Procedures

#### Production of sterile black soldier fly larvae

Test treatments varied in type, composition, and concentration of chemicals, and duration of exposure (Table 2). Series 1 consisted of five treatments (one replicate each) that have successfully achieved insect sterilization in the past (as listed in Table 1). Series 2 consisted of four treatments (three replicates each) that were designed based on the results of Series 1. Likewise, Series 3 consisted of three treatments (three replicates each) that were designed based on the results of Series 2. The experiments were conducted once. All procedures were performed using sterile techniques under a laminar flow cabinet.

**Table 2.** Sterilization treatments tested to generate sterile black soldier fly eggs (Protocol 1)^*a*^

Series	Treatment	Chemicals^*b*^ used for immersion	Concentrations (% v/v)	Duration (min)
1	1	(a) 3× NaClO	(a) 0.6	(a) 2 + 2 + 2
	2	(a) 1× NaClO (b) 2× C_2_H_5_OH	(a) 2.7 (b) 70	(a) 2 (b) 1 + 1
	3	(a) 1× NaClO	(a) 10	(a) 5
	4	(a) 1× NaClO (b) 1× C_2_H_5_OH	(a) 10 (b) 70	(a) 5 (b) 5
	5	(a) 3× C_2_H_5_OH	(a) 70	(a) 5, 1 + 1
2	6	(a) 3× NaClO	(a) 0.6	(a) 2 + 2 + 2
	7	(a) 1× NaClO (b) 2× C_2_H_5_OH	(a) 2.7 (b) 70	(a) 2 (b) 1 + 1
	8	(a) 1× NaClO (b) 1× C_2_H_5_OH	(a) 10 (b) 70	(a) 5 (b) 5
	9	(a) 1× C_2_H_5_OH (b) 1× NaClO	(a) 70 (b) 0.6	(a) 1 (b) 1
3	10	(a) 1× NaClO (b) 2× C_2_H_5_OH	(a) 2.7 (b) 70	(a) 2 (b) 1 + 1
	11	(a) 1× C_2_H_5_OH (b) 1× NaClO (c) 1× C_2_H_5_OH (d) 1× NaClO	(a, c) 70 (b, d) 0.6	(a, c) 2 (b, d) 2
	12	(a) 2× C_2_H_5_OH (b) 1× NaClO:C_2_H_5_OH = 1:1	(a) 70 (b) 10, 70	(a) 2 (b) 2

^*a*^(a), (b), (c), (d) = subsequent steps; all eggs were washed three times with sterile water after NaClO and/or C_2_H_5_OH treatments.

^*b*^NaClO = sodium hypochlorite; C_2_H_5_OH = ethanol.

Eggs were obtained from a black soldier fly colony maintained by Eawag (Dübendorf, Switzerland), according to Dortmans et al. (2017). Then, 0.1 g of eggs were transferred to a 2-ml Eppendorf tube using a metal spatula and separated with gentle stirring. They were sterilized using the treatments listed in Table 2. After each immersion in NaClO and/or C_2_H_5_OH, we discarded the supernatant before adding the next round of chemicals. After completion of all chemical washing cycles, we washed the eggs thrice with sterile water.

We incubated the eggs at 30°C for 2 wk in two different media in an incubator. One medium was thioglycollate broth (VWR, Dietikon, Switzerland) with a pH of 7.1 ± 0.2; 3 ml dispensed into 15-ml falcon tubes. It consisted (g/liter) pancreatic digest of casein, 15.0; yeast extract, 5.0; dextrose, 5.5; sodium chloride, 2.5; agar, 0.75; l-cystine, 0.5; sodium thioglycollate, 0.5; and resazurin sodium, 0.001. Another medium was Columbia blood agar (VWR) distributed in Petri dishes (diameter: 90 mm). It consisted (g/liter) of defibrinated sheep blood, 50; agar, 15; pancreatic digest of casein, 10; meat peptic digest, 5; heart pancreatic digest, 3; yeast extract, 5; sodium chloride, 5; and starch, 1.

Egg sterility, larval hatching, and growth were determined as absent/present every 4 d by visual inspection of falcon tubes and agar plates. Falcon tubes were observed for color change while agar plates were observed for possible visible colonies and larval crawling lines. Replicates were considered to be nonsterile if they appeared turbid or did not resemble eggs, egg debris, larvae, their crawling lines, or frass. Hatching was defined as the stage where larvae could be seen outside the eggs. Growth was defined as the stage when larvae had increased in size, such that they were larger than newly hatched larvae. In each series, treatments were compared with two control groups namely untreated eggs (three replicates each) and eggs exposed to three washing cycles with sterile water (three replicates each) (Brummel et al. 2004, Ridley et al. 2012).

Data were analyzed in R version 3.6.2 (R Core Team 2017). Absent/present data for egg sterility, larval hatching, and growth were normalized by the number of replicates. Means and standard deviations were calculated per treatment and control.

#### Optimizations of nutrient supply for sterile black soldier fly larvae

Even though we succeeded at egg sterilization and larval hatching (Table 2, Treatment 11), the hatched larvae did not grow in either thioglycollate broth or on Columbia blood agar. We hypothesized that this was due to a lack of essential nutrients that are usually provided by larva-associated microbes. Given the widespread applications of sterile insects, we further assumed that larval growth could be sustained by feeding them with a diet containing all essential nutrients, including those that are provided by microbes. Therefore, we tested the effect of four different diets on larval growth. Diets were prepared in glass bottles (1- to 3-liter), mixed with sterile water, and autoclaved (FOB5, Fedegari Group, Albuzzano PV, Italy) at 121°C for 15 min before distribution in Petri dishes (diameter: 90 mm). Diet 1 consisted (mg/g) of yeast (Sigma-Aldrich, Buchs, Switzerland), 500; d-glucose (Sigma-Aldrich), 100; and standard agar (VWR), 15. Diet 2 consisted (mg/g) of yeast, 250; d-glucose, 250; and standard agar, 15. Diet 3 consisted (mg/g) of yeast, 250; d-glucose, 250; and meat liver agar (Sigma-Aldrich), 34. Diet 4 consisted (mg/g) of yeast, 250; d-glucose, 250; and Columbia agar, 42. The remainder to 1,000 mg/g consisted of sterile water.

However, the autoclaved diets did not promote larval growth. Therefore, we sterilized the diets using a nonthermal treatment, namely the high-energy electron beam (HEEB) technique. Prior to HEEB treatment, diets were sealed in air-tight and water-tight polyamide-polyethylene bags up to a maximum fill line of 4 cm and then transported on ice to a commercial HEEB operator (Leoni Studer AG, Däniken, Switzerland). Diets were irradiated with 10 MeV electron beam at a dose of 32 kGy in accordance with the ISO 11137-3:2017 standard (ISO 2017), and subsequently surface-sterilized for 30 min on both sides, using the UV sterilization program of a laminar flow cabinet. Finally, diets were distributed on sterile Petri dishes (diameter: 90 mm). Sterility of the HEEB-treated diets was confirmed in triplicate after incubation at 30°C for 7 d on Columbia blood agar plates and in thioglycollate broth. Three different diets that could possibly provide all essential nutrients were treated with HEEB. The first diet was poultry feed (UFA 625, UFA AG, Herzogenbuchsee, Switzerland) where sterile water had been added, resulting in a moisture content of 600 mg/g. Poultry feed has been used as a positive control in several black soldier fly larvae growth studies (Diener et al. 2009, 2011; Nguyen et al. 2013; Cammack and Tomberlin 2017; Spranghers et al. 2017). The second diet was a liver pie (Le Parfait, Nestlé, Vevey, Switzerland). According to the manufacturer’s statement, the product contains pig liver, yeast, palm fat, maltodextrin, potato starch, salt, sunflower oil, corn starch, and extracts of flowers, herbs, and spices. Liver-based diets have also been used by Mumcuoglu et al. (2001). Liver is a rich source of B vitamins such as riboflavin that are known to promote insect growth (Storelli et al. 2011, Li and Gu 2016, Téfit and Leulier 2017). The third diet was an artificial diet previously used by Cammack and Tomberlin (2017) consisting (per g) of 440 mg cellulose, 126 mg casein, 42 mg peptone, 42 mg albumen, 105 mg sucrose, 105 mg dextrin, 5.5 μl linoleic acid, 0.55 mg cholesterol, 0.275 mg ascorbate, 0.180 mg vitamin mixture, 0.125 μl ethanol, and 25 mg Wesson’s salt mixture (all Sigma-Aldrich).

Larval hatching and growth on diets were assessed according to the methods described above by inoculating diets with 0.1 g of black soldier fly eggs (five replicates per diet) treated with Treatment 11 (Table 2). Egg sterility was assessed (five replicates per diet) according to the methods described above with thioglycollate broth and Columbia blood agar. Egg sterility, larval hatching, and growth were determined as absent/present following incubation for 10 d at 30°C. Data were analyzed as described above for the production of sterile black soldier fly larvae.

### Results and Discussion

Protocol 1 describes the production of sterile, viable, and growing black soldier fly larvae. Only Treatment 11 listed in Table 2 resulted in eggs that successfully hatched into viable larvae (Table 3). These larvae grew on all three HEEB-treated diets (poultry feed, liver paste, and artificial diet), but not on the autoclaved diets (Table 4).

**Table 3.** Effect of sterilization treatments (Protocol 1) on egg sterility, larval hatching, and growth determined by inoculation of eggs in thioglycollate broth and on Columbia blood agar

Series	*n*	Treatment	Egg sterility (% of replicates)	Larval hatching (% of replicates)	Larval growth (% of replicates)
1	1	1	0	n.d.	100
	1	2	0	n.d.	100
	1	3	0	100	100
	1	4	0	100	100
	1	5	0	100	100
	1	Untreated	0	100	100
	1	H_2_O control	0	100	100
2	3	6	0	100	100
	3	7	33	100	33 (nonsterile replicates)
	3	8	33	100	66 (nonsterile replicates)
	3	9	0	100	100
	3	Untreated	0	100	100
	3	H_2_O control	0	100	100
3	3	10	0	100	66
	3	11	100	100	0
	3	12	0	100	66
	3	Untreated	0	100	100
	3	H_2_O control	0	100	100

*n* = number of replicates; n.d. = not detected.

**Table 4.** Effect of various diets in combination with diet sterilization treatments on larval sterility, hatching, and growth

Diet	Sterilization treatment	*n*	Larval sterility (% of replicates)	Larval hatching (% of replicates)	Larval growth (% of replicates)
Artificial diet 1	Autoclave	5	100	n.d.	0
Artificial diet 2	Autoclave	5	100	n.d.	0
Artificial diet 3	Autoclave	5	100	n.d.	0
Artificial diet 4	Autoclave	5	100	n.d.	0
Poultry feed	HEEB	5	100	100	40
Liver pie	HEEB	5	100	100	60
Artificial diet	HEEB	5	100	100	40
Untreated	Untreated	5	0	100	100
H_2_O control	Untreated	5	0	100	100

Eggs used were sterilized with Treatment 11 (listed in Table 2) (Protocol 1). *n* = number of replicates; n.d. = not detected.

Our protocol differs from the established methods for *Drosophila* spp. (Table 1). Unlike *Drosophila* spp., the immersion of black soldier fly eggs in sodium hypochlorite for several minutes did not result in sterilization. The successful treatment involves two cycles, each starting with ethanol (2 min), followed by sodium hypochlorite (2 min). These differences could be due to species differences in egg size, physiology, and/or microbial load and composition.

Our results also suggest that microbes are important for growth of black soldier fly larvae. In contrast to *Drosophila* spp., sterile black soldier fly larvae did not grow on autoclaved diets (Table 4), while nonsterile black soldier fly larvae did (Table 3). Growth of sterile black soldier fly larvae was observed in 40% of the replicates of HEEB-treated poultry feed and artificial diet, and in 60% of the replicates of HEEB-treated liver pie diet. The eggs used for these tests had been subjected to Treatment 11 (Table 2). They were found to be sterile based on visual assessment following incubation in thioglycollate broth and on Columbia agar. However, the lack of larval growth in some of the replicates likely indicates that even Treatment 11 may have been damaging to some extent. Although we cannot rule out the possibility that some essential nutrients had been lacking in the four autoclaved artificial diets; results of our growth tests more likely suggest that sterile black soldier fly larvae need nutrients that are inactivated during autoclaving. Essential heat-sensitive compounds such as bioactive peptides or vitamins might be better preserved by HEEB treatment rather than autoclaving. Unlike *Drosophila* larvae that grow well under axenic conditions on several autoclaved diets (Brummel et al. 2004, Ridley et al. 2012, Newell and Douglas 2014, Koyle et al. 2016, Kim et al. 2018), other insect species such as *Musca domestica* (Schmidtmann and Martin 1992, Watson et al. 1993) and *Musca autumnalis* (Diptera: Muscidae) (Hollis et al. 1985) were found to be strongly dependent on the presence of microbes for their growth. Cohen (2015) suggested that larva-associated microbes synthesize essential nutrients (e.g., vitamins, amino acids, signaling molecules, and sterols) that may otherwise be lacking in insect diets. Therefore, these insect species are unable to grow when sterilized.

### Proof of Concept and Limitations

We demonstrated that a four-step treatment with alternating immersions of ethanol and sodium hypochlorite can produce sterile and viable black soldier fly larvae that can possibly be used as a research model system. Our HEEB-treated diets then facilitated the growth of sterile larvae, while our autoclaved diets did not. These results indicate that there are still unknown micronutrients that affect larval growth. Also, certain metabolites that may be normally synthesized by microbes are essential for larval growth. However, the survival and hatching of sterilized black soldier fly eggs may not depend on these factors.

Several limitations should be considered when interpreting our results and their applications in future research.

i) Tests were conducted with few biological replicates (i.e., 3–5) in one experiment.
ii) Compared to visual identification of microbial growth in the thioglycollate broth and Columbia agar plates, microbial gene expression by RNA sequencing (Brummel et al. 2004, Ridley et al. 2012) would have provided more conclusive evidence that sterile insects were free of microbial contamination.
iii) In future, all test diets should be autoclaved at the same time as HEEB treatment, to unambiguously identify whether it is the sterilization process or differences among diets that primarily affects the growth of sterile black soldier fly larvae.
iv) Growth of sterile black soldier fly larvae on HEEB-treated diets was not reliable. Unintentional microbial contamination may have contributed to the growth of sterile black soldier fly larvae. Therefore, mixing antibiotics in their diets may prevent this problem (Sharon et al. 2010, Heys et al. 2018).
v) Growth of sterile larvae was much slower compared to nonsterile ones. However, this was determined on the basis of visual observations, which can be subjective. Therefore, in future experiments, increasing the experimental duration will allow determination of larval weight and thus more conclusive results.

## Protocol 2: Frass Collection to Study Black Soldier Fly Larvae Digestion

### Experimental Design and Procedures

**Fig. 1.**
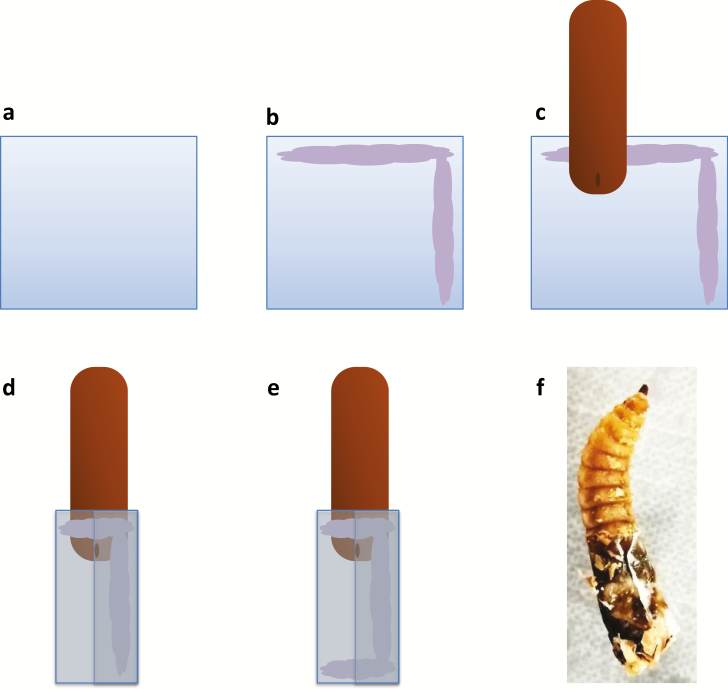
Method to attach frass collection device to black soldier fly larvae: (a) square cut from plastic sheet, side length approximately 1.5 times the larval diameter; (b) application of instant glue; (c) attaching to larval rear, as low as possible to allow movement, yet high enough to enable frass excretion; (d) plastic sheet rolled around larvae and sealed; (e) sealing bottom end of plastic sheet with glue; (f) black soldier fly larvae larva with collected frass, following feeding for 24 h on poultry feed and separation from the residue.

We developed a novel device to collect black soldier fly larvae frass and assessed its impact on larval behavior and reliability of frass collection. Squares with a side length of approximately 1.5 times the diameter of an individual larva were cut from thin plastic bags (Aromata, Lidl, Switzerland) (Fig. 1a). Ultra Gel glue (Pattex, Henkel & Cie. AG, Pratteln, Switzerland) was applied to two adjoining edges of the plastic square (Fig. 1b). The larva was then placed onto the square such that the top edge of the square was aligned horizontally with the middle of the second and third bottom segments (Fig. 1c). The plastic sheet was then rolled up and sealed (Fig. 1d). Finally, glue was applied to the bottom edge of the plastic sheet to close the device (Fig. 1e). We tested the device on 15- to 18-d-old larvae from the colony and the poultry feed (both mentioned in Protocol 1). After separating the residue, we attached the frass collection device to 25 larvae. Larval weight was then recorded with a precision laboratory scale. We used a light microscope to confirm that the frass collection device was fitted correctly. Another 25 larvae without a frass collection device served as the control group. Larvae were then placed in a plastic container with the poultry feed moistened to 600 mg water/g of feed. Feed was provided at a rate of 250 mg wet weight/larva per day which likely exceeded the ad libitum intake. After feeding for 24 h in a climate chamber (HPP 260, Memmert GmbH, Schwabach, Germany) at 28°C and 75% RH, larvae were separated from the remaining feed residue (Fig. 1f), washed with tap water, and dried with paper towels. Next, the collection devices attached to larvae were opened at the bottom and frass was gently removed using a small metallic spatula, transferred to a tared 2-ml Eppendorf tube, and weighed with a precision balance (BM-65, Phoenix Instrument, Garbsen, Germany). Finally, larvae were weighed with their empty collection device. Larval weight (mg wet weight) was calculated by subtracting the weight of the empty collection device from the larval weight with the empty collection device. Diet reduction (mg wet weight) was calculated by subtracting the initial diet weight. As the feeding period was 1 d, this was equivalent to the daily diet reduction.

Samples of the diet (poultry feed), frass collected by the developed device, and larval gut homogenate from the control group were analyzed for metabolites associated with insect and microbial digestion (Cohen 2015, Kim et al. 2018). Similar to the method used for black soldier fly larvae by Bonelli et al. (2019) the larval gut homogenate sample was prepared using dissecting scissors by removal of the chitin, fat body, and trachea surrounding the gut from three larvae. We analyzed glucose, galactose, citrate, succinate, lactate, formate, acetate, propionate, and ethanol using high-performance liquid chromatography (HPLC). Before injection into the HPLC system, 100 mg of the sample was transferred into a 2-ml Eppendorf tube. Then, 300 µl of 10 mM sulphuric acid and a glass bead were added. Samples were vortexed until homogenous and centrifuged at 16,000 × *g* at 4°C for 10 min, and then 150 µl of the supernatant was filtered through a 0.45-µm nylon membrane (Infochroma AG, Zug, Switzerland) into a conical flask insert. The HPLC system consisted of a VWR Hitachi Chromaster 5450 RI-Detector using a Rezex ROA-Organic Acid (4%) precolumn connected to a Rezex ROA-Organic Acid (8%) column, equipped with a Security Guard Carbo-H cartridge (4 × 3.0 mm). Sample volumes of 40 μl were injected into the HPLC with a flow rate of 0.6 ml/min at a constant column temperature of 80°C using a mixture of sulfuric acid (10 mM) and sodium azide (0.05 g/liter) as eluent. Concentrations were determined using external standards via comparison of the retention time. Peaks were integrated using the EZChromElite software (Version V3.3.2 SP2, Hitachi High-Tech Science Corporation, Tokyo, Japan). Limit of detection was defined as >0.8 mM.

Data were analyzed in R version 3.6.2 (R Core Team 2017). Means and standard deviations of larval weight and diet reduction were calculated.

### Results and Discussion

This is the first study to develop a device to collect frass from black soldier fly larvae and analyze it for the presence of metabolites that are produced during microbial fermentation.

The frass collection device did not impede larval development. Mean ± standard deviation of larval weight for the group with the collection device was 234 mg ± 15 at the beginning of the experiment and 259 mg ± 26 at the end of the experiment (*n* = 24, one larva with a weight gain of 101 mg was considered as an outlier). For the control group (*n* = 25) larval weight at the beginning was 239 mg ± 16 and at the end was 248 mg ± 20. Diet reduction (in wet weight) was 66 mg/d per larva in the group with the collection device and 56 mg/d per larva in the control group. During the 24-h period of larval feeding, 194 mg frass from 25 larvae was collected using the device, resulting in an average of 7.8 mg frass per larva. Some amount of frass could not be collected, as it was stuck to the plastic material, and removing it with water would have interfered with the determination of total frass mass and downstream analyses of metabolites.

Our novel collection method facilitated the analysis of black soldier fly larvae frass and comparison of the nutrient and metabolite composition of degraded diet to that of larval diet and larval guts. We detected a concentration gradient for the different metabolites (Table 5). The concentration of metabolites associated with the diet nutrients (glucose and galactose) was generally higher in the diet compared to larval guts, suggesting nutrient digestion in the larval digestive tract. Interestingly, all metabolites associated with microbial fermentation (succinate, lactate, formate, acetate, propionate, and ethanol) were more concentrated in frass than in either diet or guts. A higher concentration of these metabolites in frass could be explained by catabolic activity of enzymes either in the larval digestive tract or by microbes. However, since the concentrations of the commonly known carbohydrate degradation pathway metabolites lactate and acetate were higher in frass compared to larval gut content, microbial activity appears more plausible. Bacterial strains producing lactate and acetate, namely *Lactobacillus* and *Acetobacter*, respectively, are known to occur in gut microbiota of *Drosophila* (Storelli et al. 2011, Téfit and Leulier 2017). Further, several *Lactobacillus* strains are also prevalent in black soldier fly larvae microbiota (Jeon et al. 2010, Bruno et al. 2019).

**Table 5.** Metabolites (µmol/g) detected in poultry feed, larval gut homogenate (composite of three larvae guts), and larval frass (composite from 25 larvae) (Protocol 2)

Compound	Poultry feed	Larval gut homogenate	Larval frass
Glucose	30.7	12.4	182.3
Galactose	20.3	1.0	6.4
Citrate	8.3	0.8	0
Succinate	1.1	1.6	6.5
Lactate	3.2	3.2	183.1
Formate	11.6	3.9	18.0
Acetate	1.8	2.7	65.6
Propionate	2.2	2.7	4.7
Ethanol	0	3.9	18.8

### Proof of Concept and Limitations

We developed a method to collect frass from individual black soldier fly larvae and determined the composition of nutrients and metabolites in black soldier fly larvae frass for the first time. In large-scale black soldier fly larvae rearing, such a device will help determine the digestibility of various organic wastes and side streams, using mass balance calculations between dietary nutrients and frass. It will also help identify the specific nutrient requirements of black soldier fly larvae. This knowledge could increase the efficiency and reliability of rearing performance, which is a key determinant of the sustainability of insect larval biomass as raw material, in food and animal feed markets.

Although promising, this protocol has several limitations that should be considered in future research:

i) Our frass collection device needs to be attached and removed manually, which is impractical for a large number of larvae. To determine insect nutrient requirements, a large sample size (e.g., >1,000) is needed. Otherwise, most nutrients cannot be analyzed (one exception might be N content with micro-Kjeldahl).
ii) Probable sources of error include overestimation of larval weight due to incomplete emptying of the collection device. Also, water can accumulate between the plastic material and body of the larva, thus, possibly altering the larval moisture content.
iii) Frass underestimation is possible if the method is implemented as described, due to the incomplete emptying of collection devices. This can be overcome by flushing both larvae and the collection device with water, and drying them. Alternately, even incomplete frass samples can be used to determine digestibility, by mixing indigestible markers like alkanes (Mayes et al. 1986) into the diet. The proportion of diet that disappears (is digested) in the gut can be calculated from the increase in marker concentration between diet and frass.
iv) Considering the long storage time of frass in the collection device (up to 24 h) and high temperature (28°C), it is possible that the change in quantity and composition of nutrients, metabolites, and microbes after excretion is partially due to decomposition of digestible nutrients outside of the larval gut.
v) Alternatives to our frass collection device should also be considered. These include larval dissection and extraction of the midgut content as described by Bonelli et al. (2019). Posterior midgut or hindgut content is likely similar to frass. Additionally, Storelli et al. (2018) suggested feeding *D. melanogaster* larvae with a blue dye, bathe them overnight in phosphate-buffered saline (PBS), determine the frass quantity from the absorbance of the blue dye in PBS, and use the suspension for analyses of nutrients and metabolites.
vi) To verify whether bacterial metabolism is responsible for metabolite production in the frass, DNA or RNA sequencing can be conducted on the diet, gut content, and frass.

### Conclusions

The present study describes novel methods for the generation of sterile black soldier fly larvae, gently sterilized larval diets, and a device to collect black soldier fly larvae frass. These methods could be used in research on host–microbe interactions and larval nutrient requirements. Results from such studies may possibly improve process performance and reliability in large-scale black soldier fly larvae rearing. These methods are therefore valuable to the emerging insect industry, for the production of raw material for food and animal feed markets. Our methods need to be further refined and tested.
